# Common Coinfections of *Giardia intestinalis* and *Helicobacter pylori* in Non-Symptomatic Ugandan Children

**DOI:** 10.1371/journal.pntd.0001780

**Published:** 2012-08-28

**Authors:** Johan Ankarklev, Elin Hestvik, Marianne Lebbad, Johan Lindh, Deogratias H. Kaddu-Mulindwa, Jan O. Andersson, Thorkild Tylleskär, James K. Tumwine, Staffan G. Svärd

**Affiliations:** 1 Department of Cell and Molecular Biology, BMC, Uppsala University, Uppsala, Sweden; 2 Centre for International Health, University of Bergen, Bergen, Norway; 3 Department of Diagnostics and Vaccinology, Swedish Institute for Communicable Disease Control, Solna, Sweden; 4 Department of Microbiology, School of Biomedical Sciences, Makerere University, Kampala, Uganda; 5 Department of Paediatrics and Child Health, School of Medicine, College of Health Sciences, Makerere University, Kampala, Uganda; Georgetown University, United States of America

## Abstract

**Background:**

The protozoan parasite *Giardia intestinalis* and the pathogenic bacterium *Helicobacter pylori* are well known for their high prevalences in human hosts worldwide. The prevalence of both organisms is known to peak in densely populated, low resource settings and children are infected early in life. Different *Giardia* genotypes/assemblages have been associated with different symptoms and *H. pylori* with induction of cancer. Despite this, not much data are available from sub-Saharan Africa with regards to the prevalence of different *G. intestinalis* assemblages and their potential association with *H. pylori* infections.

**Methodology/Principal Findings:**

Fecal samples from 427 apparently healthy children, 0–12 years of age, living in urban Kampala, Uganda were analyzed for the presence of *H. pylori* and *G. intestinalis*. *G. intestinalis* was found in 86 (20.1%) out of the children and children age 1<5 years had the highest rates of colonization. *H. pylori* was found in 189 (44.3%) out of the 427 children and there was a 3-fold higher risk of concomitant *G. intestinalis* and *H. pylori* infections compared to non-concomitant *G. intestinalis* infection, OR = 2.9 (1.7–4.8). No significant association was found in the studied population with regard to the presence of *Giardia* and gender, type of toilet, source of drinking water or type of housing. A panel of 45 *G. intestinalis* positive samples was further analyzed using multi-locus genotyping (MLG) on three loci, combined with assemblage-specific analyses. *Giardia* MLG analysis yielded a total of five assemblage AII, 25 assemblage B, and four mixed assemblage infections. The assemblage B isolates were highly genetically variable but no significant association was found between *Giardia* assemblage type and *H. pylori* infection.

**Conclusions/Significance:**

This study shows that *Giardia* assemblage B dominates in children in Kampala, Uganda and that the presence of *H. pylori* is an associated risk factor for *G. intestinalis* infection.

## Author Summary

*G. intestinalis* and *H. pylori* are known to infect the gastrointestinal tract of humans early in life and to be very prevalent in endemic areas throughout life. *H. pylori* colonizes the gastric mucosa and may give rise to peptic ulcers, chronic gastritis and gastric cancer whereas *Giardia* causes diarrhea, bloating, flatulence and malnutrition. The genetic variability within *G. intestinalis* is high with two genotypes or assemblages (A and B) infecting humans. These two different genetic types of humans have also been associated with differences in symptoms. Here we have studied these two infections in non-symptomatic children in Kampala, Uganda. *H. pylori* was found in 44% out of the 427 children and *G. intestinalis* was found in 20% with children age 3<6 years showing the highest rates of colonization. The children were primarily infected with Giardia assemblage B parasites and *Giardia* infected children had a 3-fold higher risk of also having *H. pylori* infection. However, this was independent of *Giardia* assemblage type. This information will be important in the development of new control measures of these prevalent pathogens in Uganda and other low-income countries.

## Introduction

In low-income countries co-infections involving several different pathogens commonly ocurr [1]. Several recent, cross-sectional studies from different locations, have reported a potential association between *G. intestinalis* and *H. pylori* [2], [3], [4]. Both organisms colonize the gastrointestinal tract in their human hosts within a close proximity and both organisms are known to infect children at a high rate in low-income countries [5], [6], [7].

*H. pylori* is a gram-negative bacterium that is estimated to infect approximately half of the world population. It colonizes the gastric mucosa of its human host where it may give rise to symptoms such as recurrent peptic ulcers and chronic gastritis, and has also been associated with gastric cancer [8]. The prevalence of *H. pylori* is high in low-income countries and it was recently shown to colonize 46% of children age 1<3 years in an area of urban Kampala, Uganda [5].

The protozoan parasite *G. intestinalis* (syn. *G. lamblia, G. duodenalis*) is the causative agent of giardiasis in a wide range of vertebrates, including humans. The parasite is estimated to cause 280 million cases of human giardiasis per year [9]. The disease is characterized by bouts of diarrhea, bloating, flatulence and malnutrition, and is especially troublesome in children living in low-income countries where stunted growth and poor cognitive function have been correlated with the disease [9], [10]. Asymptomatic *Giardia*-infections are common [11], where the host may act as a reservoir for transmission of the disease. Eight different *G. intestinalis* genotypes or assemblages have been described (A-H) [12], where assemblages A and B infect humans and other mammals and assemblages C through H are more host-specific [13]. Recent data suggest that *Giardia* assemblage A and B can actually be two different species [14] and several studies have recently shown associations between assemblage type and specific symptoms [15], [16], [17].

To date, a large number of human *Giardia* samples from Europe, Australia, South-America and Asia, have been characterized on the molecular level, mainly on one, but also several genetic loci. Studies dealing with genetic characterization of human-infecting *Giardia* in the sub-Saharan regions of Africa, are however, much more scarce [18], [19]. One of these studies was performed in rural western Uganda and an average *Giardia* prevalence of 40% was detected [19]. Genotyping using the rSSU-rDNA gene showed that the distribution between assemblage A and B was even (53% A and 47% B, [19]). Although the occurrence of *Giardia* is assumed to be common in Kampala, Uganda, there are not much data available to confirm this. Also, the prevalence of different *Giardia* assemblages in infected individuals has not yet been investigated in this area.

The aim of this study was to to assess a potential correlation between certain *G. intestinalis* assemblages and concomitant infection of *H. pylori* in apparently healthy children aged 0–12 years from Kampala, Uganda using multi-locus genotyping.

## Methods

### Study design

This study was part of a survey that was carried out in October/November 2007 in Kampala, Uganda. Details regarding the set up of the study are described in Hestvik et al. [5], [20].

### Study area

Sampling of patient fecal material was carried out in Mulago II parish in Kampala, Uganda. Kampala is located just north of the equator and has a tropical, humid climate with two rainy seasons (mid February – Mid May and September - December) and two drier periods in between. This is a resource limited area of the town, characterized by informal settlements, congested living, lack of proper sanitation conditions and low education level among adults but it is supported with tap water by Plan International [5].

### Source of isolates

All samples originated from a completed *H. pylori* survey on apparently healthy children where 427 fecal samples were analyzed [5]. Children aged 0–12 years were recruited after door-to-door visits; an equal number of children in each age category of 0<1 year, 1<3 years, 3<6 years, 6<9 years and 9<12 years (around 85 per age group). Participants were included in the study if: 1) they were apparently healthy, 2) aged between 0<12 years, 3) had an informed consent from caretaker and 4) were able to produce a stool sample within three consecutive days.

### Ethics approval

Ethical approval was obtained from Makerere University, Faculty of Medicine, Research and Ethics Committee in Uganda, the Regional Committee for Medical and Health Research Ethics, West-Norway (REK-VEST, Ref. Nr. 2007/13898-ANØL) and the regional ethical committee at Uppsala University, Uppsala Sweden (Ref. Nr. 2009/025). The data collectors were trained in ethical issues prior to the study. Oral and written information about the study was given to the caretakers (parents/guardians) either in English or a preferred local language. Informed consent in writing was obtained from at least one caretaker (parent/guardian) of each of the study participants.

### Sample diagnostics

A stool sample was requested from each participating child and was collected in air-tight containers either at time of the encounter, at the end of the day, or the following morning. Stool samples were transported from the field to the laboratory at ambient temperature twice daily and stored in a +4°C fridge until the same afternoon or the following day when analysis were carried out. All stool samples were investigated by microscopy for protozoa and helminths, a culture was performed to assess for enteropathogens and all samples were tested for *Helicobacter pylori*. The presence of *H. pylori* antigen in feces was evaluated using HpSA ImmunoCardSTAT as described in Hestvik et al [5].This faecal monoclonal antigen test has high sensitivity, specificity, and accuracy in children, 91–96%, 95–96% and 94–96%, respectively [21], [22] Instructions given by the manufacturer were followed and all positive control tests were positive. The results were reported as positive or negative on the basis of the manufacturer's cut-off values. Furthermore, *Giardia* cysts and trophozoites were identified on wet fecal smears using light microscopy and subsequently fixed in ethanol as previously described [23]. Human fecal samples containing *Giardia* cysts (n = 86) were collected. The DNA content of the *Giardia* cysts was studied using FITC labelled CWP (cyst-wall protein) -specific antibodies (Agua-Glo, Waterborne Inc., New Orleans, LA, USA) together with DAPI (4′6-diamino-2-phenyl-indole) prior to the extraction of the DNA. DNA from the 45 samples staining strongly positively with DAPI, indicating intact DNA, was extracted in Sweden as described elsewhere [23].

### PCR and sequencing

All PCR primers used in this study can be seen in Supplementary File S1. Nested PCR was performed to amplify a 511 bp fragment of the ß-giardin gene (*bg*), a 530 bp fragment of the triose phosphate isomerase gene (*tpi*), and semi-nested PCR was used to amplify a 430 bp fragment of the glutamate dehydrogenase gene (*gdh*) [24], [25], [26]. Samples that were identified as assemblage AII, were further analyzed on loci in chromosome 3 (ORFs: GL_50803_113553 and GL_50803_3095) and chromosome 5 (ORF: GL_50803_39587) according to Cooper et al. [27], [28]. All PCR products were analyzed using electrophoresis on 1.5% agarose gels stained with GelRed (Biotium, Hayward, CA, USA). Positive PCR products amplified with primers from the respective loci were sequenced in both directions using the BIG DYE 3.1 sequencing kit (Applied Biosystems, La Jolla, CA, USA). Prior to sequencing, the PCR products were purified using Exo-sap IT™ according to the manufacturer's instructions (GE Healthcare, Uppsala, Sweden). Post sequencing, the labelled products were purified using the Qiagen Dye-ex 2.0 spin kit (Qiagen, Hilden, Germany). The sequenced products were analyzed in an Applied Biosystems 9100 Seq (Applied Biosystems, La Jolla, CA, USA). Sequences and chromatogram were analyzed and edited using the BioEdit software, Version 7.0.5. BLAST analyses were performed on all sequences (http://www.ncbi.nlm.nih.gov/blast/), and unique sequences were uploaded in GenBank. Sequence data is found in Supplementary Files S2, S3, and S4.Assemblage A and B specific primers, targeting assemblage-specific regions of the *tpi* gene [29], [30], were utilized to detect mixed infections.

### Phylogenetic analysis

The nucleotide sequence datasets for the *bg*, *gdh* and *tpi* genes used in a previous study [16] were used as references in the phylogenetic analyses. Unambiguous gene sequences (i. e. sequences lacking double peaks) obtained in this study were added to reference datasets. This yielded datasets of 475, 393, and 490 nucleotide positions for *bg, gdh* and *tpi*, respectively, suitable for phylogenetic analyses. RAxML version 7.0.4 [31] was used to perform maximum likelihood analyses with the GTR substitution model and among-site rate variation (GTRGAMMA), together with bootstrap analyses with 500 replicates. The sequences from the chromosome 3 locus listed in Supplementary Table S3 were analysed using the same methodology.

### Nucleotide sequence accession numbers

The Assemblage B nucleotide sequences obtained in this study, that were unique compared to other *Giardia* sequences submitted to public databases, have been deposited in Gen Bank under the following accession numbers [GenBankAcc No: JQ303244–JQ303248].

### Statistical methods

To explore the prevalence of *Giardia* and factors associated, binary logistic regression as well as multiple logistic regression were performed. Factors with p-values higher than 0.1 were not included in the final model.

## Results

### Clinical data and observations

**Figure 1:**
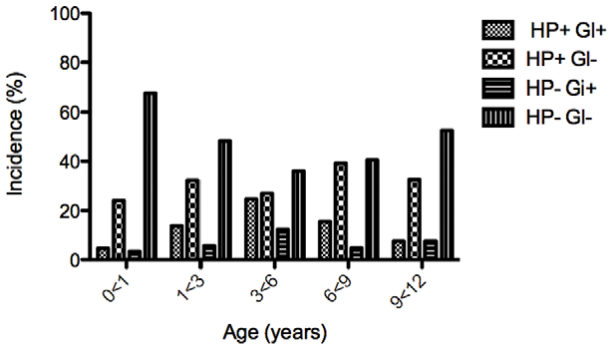
Incidence of G. *intestinalis* and *H. pylori* infections in children of different ages.

Baseline characteristics of the whole study population (n = 427), are presented in the Methods section and in Hestvik et al [5]. *Giardia* cysts or trophozoites were observed in fresh stool by direct light microscopy in samples from 86 children. In these 86 children the mean age (±SD) was 5.0 (3.1) years: for girls 5.3 (3.3) years and boys 4.5 (2.8) years (Table 1). The genders were equally represented: 41 (47.7%) girls and 45 (52.3%) boys. The prevalence of *G. intestinalis* was 20.1% (86/427) and peaked in the age group including children 3<6 years, 28.7%. There was a significantly higher risk of colonization in the older age groups (1–12 years) compared to the youngest (0 to 1 years, Table 1). There was no statistically significant association between *G. intestinalis* and gender, type of toilet, drinking water source and type of housing for the child (Table 1). Children colonized with *G. intestinalis* had a statistically significant higher risk for co-colonization with *H. pylori* (Table 1). The finding remained significant also after adjustment for age, gender, type of toilet, drinking water source and type of housing for the child OR (95%CI) 2.8 (1.3–6.2). The prevalence of *H. pylori* was already 29% in the youngest age group 0<1 year, peaking in the 6<9 age group with 55% [5], (Fig. 1). This should be compared to an 8% *Giardia* prevalence in the youngest group and a peak in the 3>6 age group (Fig. 1). Thus, the level of *Giardia* colonization starts off slower but peaks earlier compared to the *H. pylori* colonization.

**Table 1:** *Giardia intestinalis* infection and associated factors present in apparently healthy children from Kampala, Uganda.

	Number	*G. intestinalis* positive N (%)	Unadjusted Odds Ratio (95% Confidence interval)	Adjusted Odds Ratio* (95% Confidence interval)
**Age**				
0<1 year	88	7 (8.0)	1	1
1<5 years	150	43 (28.7)	4.7 (2.0–10.9)	3.9 (1.7–9.4)
5<12 years	189	36 (19.0)	2.7 (1.2-6-4)	2.2 (0.9–5.3)
***H. pylori*** ** colonization**				
No	238	29 (12.2)	1	1
Yes	189	57 (30.2)	3.1 (1.9–5.1)	2.9 (1.7–4.8)
**Sex**				
Male	211	45 (21.3)	1	-
Female	216	41 (19.0)	1.2 (0.7–1.9)	
**Using pit latrine**				
Yes	412	83 (20.1)	1	-
No	15	3 (20.0)	1.0 (0.3–3.7)	
**Drinking water**				
Unprotected source	384	77 (20.9)	1	-
Public tap	43	9 (20.1)	0.9 (0.4–2.1)	
**Type of housing**				
Semi-permanent	245	54 (22.0)	1	-
Permanent	182	32 (17.6)	1.3 (0.8–2.2)	

* Adjusted for age, gender, *H. pylori* colonization, type of toilet, source of drinking water and type of housing.

### Molecular analysis of *Giardia intestinalis*

Out of the panel of 45 *Giardia* samples available for molecular analysis, 31 (70%) showed the expected 511 bp *bg* fragment, 34 (76%) showed the expected 430 bp *gdh* fragment, and 29 (65%) showed the expected 530 bp *tpi* fragment when analyzed using agarose gel electrophoresis of the PCR products. Thus, a maximum of 76% of the *Giardia* positive samples could be verified when analyzed with PCR. In all cases where the PCR gave negative results, the *Giardia* cysts did not stain as strongly with DAPI, indicating that the DNA had been too degraded, thus explaining the negative PCR result.

### Sequencing at the bg locus

Products from the 32 positive PCR products at the *bg* locus yielded five assemblage A, 26 assemblage B and one mixed assemblage infection. Within assemblage A, all samples were of the AII sub-assemblage. Within assemblage B nine sequences were non-heterogeneous, out of these, four were unique and deposited in GenBank (JQ303244–JQ303247). Three sequences were identical to Sweh095 (HM165221), one was identical to Sweh003 (HM165209), and one was identical to Sweh023 (HM165212). The remaining 15 sequences had one to six heterogeneous substitutions over a total of 19 different positions at the *bg* locus. At the *bg* locus, all nucleotide substitutions, with the exception of one isolate, were present at the third coding position, indicating the absence of non-synonymous amino acid changes (Supplementary File S2).

### Sequencing at the gdh locus

Out of the 34 positive PCR products on the *gdh* locus, five were assemblage A, 28 were assemblage B and one was a mixed assemblage infection. The samples that indicated assemblage A and mixed infection at the *bg* locus showed the same results at the *gdh* locus. All assemblage A samples were of the AII sub-assemblage. Samples that were identified as assemblage B gave rise to 26 sequences with two to 14 heterogeneous substitutions over a total of 37 positions at the *gdh* locus. Three sequences were without heterogeneous substitutions, where one was identical to RW04 (AB638286) and one was identical to Sweh035 (HM136889). One sequence was unique and deposited in GenBank [Acc No: JQ303248]. At the *gdh* locus all nucleotide substitutions, with the exception of one isolate, were present at the third coding position, indicating the absence of non-synonymous amino acid changes (Supplementary File S3).

### Sequencing at the tpi locus

Sequencing at the *tpi* locus yielded three assemblage A, 25 assemblage B and one mixed infection. The same samples that indicated assemblage A and mixed infection at the two previously described loci showed the same results at the *tpi* locus, with the exception that only three out of the five assemblage A samples gave positive PCR results. All assemblage A samples were of the AII sub-assemblage. Sequencing of the assemblage B samples at the *tpi* locus generated 23 sequences with two to 14 heterogeneous substitutions over a total of 44 positions. Three sequences were without heterogeneous substitutions, where one was identical to Ba7 (EU272153), and the other two were identical to Sweh136 (HM140720. All assemblage B sequences without heterogenous substitutions were included in phylogenetic analysis, performed independently for each genetic locus.

At the *tpi* locus two non-synonymous amino acid substitutions had occurred that resulted in a stop codon in the amino acid sequence (GU1157 and GU1161), one non-synonymous substitution that had occurred in the majority of the assemblage B isolates when aligned to the GS reference strain was a substitution from a Tyr to a His (Supplementary File S4). Also several positions, where substitutions in the nucleotide sequences yielded double peaks in the chromatograms, implied potential non-synonymous amino acid substitutions (n = 16), one of which was present in a position that would lead to a stop codon in one of the isolates (Supplementary File S4).

### Verification of mixed assemblage infections using PCR with assemblage-specific primers

All 45 samples that were subjected to molecular analyses, were further analyzed using nested PCR of the *tpi* locus, where the second sets of primers are designed to be A- or B-assemblage specific. Only the 34 samples that previously indicated positive results in the PCR reactions indicated positive results when assayed with the assemblage-specific PCR (Supplementary Table S1). Out of the 34 samples, a total of four samples indicated positive results with both primer pairs, indicating mixed assemblage infection (Supplementary Table S2). Thus, four out of the 34 PCR positive samples contained both assemblage A and B *Giardia*. In summary, five out of 34 (14.7%) samples yielded assemblage AII, 25 out of 34 (73.5%) yielded assemblage B, and four out of 34 (11.8%) yielded mixed assemblage AII and B infection (Supplementary Table S2).

### Phylogenetic analysis of *G. intestinalis* at the bg, gdh and tpi loci

**Figure 2:**
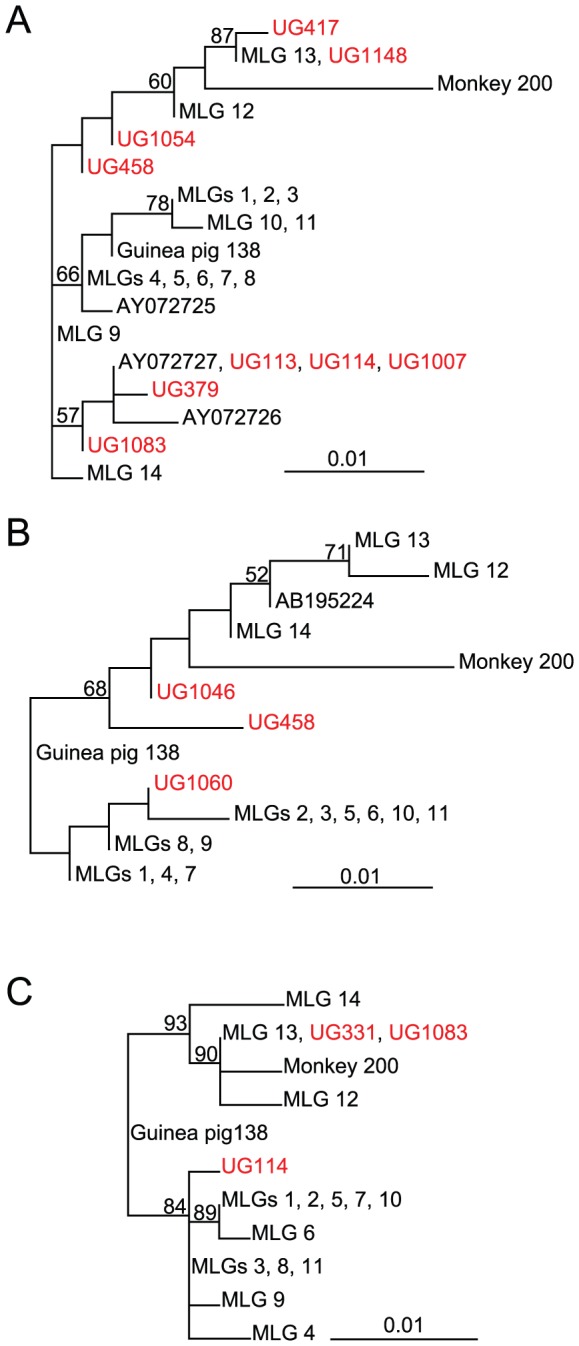
Nucleotide maximum likelihood trees based on *bg*, *gdh*, and *tpi* gene sequences from assemblage B isolates. Unambiguous sequences identified in this study were combined with isolates from our previous studies [13], [16]. Phylogenetic trees of (A) *bg*, (B) *gdh*, and (C) *tpi* gene sequences. Sequences from this study are indicated in red. Only bootstrap support values >50% are shown.

Phylogenetic analyses were performed to examine the within assemblage diversity of the obtained isolates. Sequences lacking double peaks were included in the analyses due to the uncertainty of the origin of the sequence heterogeneity within templates. The diversity of the assemblage B sequences obtained was larger with seven, three and two distinct subtypes for the *bg*, *gdh* and *tpi*, respectively (Fig. 2). For reference, sequences obtained in previous studies from our laboratory were included [13], [16]. Unfortunately, none of the isolates yielded unambiguous sequences in all three genes. Therefore, phylogenetic trees based on the individual genes are presented (Fig. 2). The Ugandan sequences are found in different parts of the assemblage B trees, most clearly in the bg tree (Fig. 2A). This suggests that the genetic diversity of the *Giardia* assemblage B lineages present in our study area in Uganda is comparable with the total diversity so far observed in humans and animals in different places in the whole world [13], [16]. Thus, the genetic variability in assemblage B *Giardia* is extremely high in this small geographical area. It is also obvious that the topologies of the three phylogenetic trees (Fig. 2A to C) do not agree. Certain isolates, e.g. UG1083, show up in different parts of the trees (Fig. 2), suggesting different evolutionary history of the different genes in one isolate. This could be due to recombination between the different assemblage B isolates.

### Sequencing of assemblage A isolates at two additional AII typing loci

**Figure 3:**
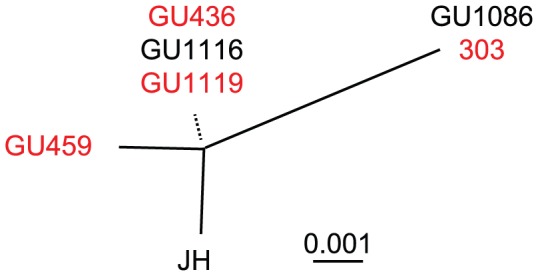
Nucleotide maximum likelihood tree of the Chromosome 3 locus from the assemblage A isolates. Phylogenetic relationship of the sequences listed in Supplementary Table S3. Dotted line indicates the branching position of three sequences with a very short branch length. Sequences in red and black differ by four SNPs in the chromosome 5 locus (Supplementary Table S4).

The five assemblage A isolates were sequenced on the *bg*, *gdh* and *tpi* loci and they were all identical to sequences previously classified as MLG AII-2, earlier detected in 26 Swedish human isolates [16]. In order to increase the resolution of the genotyping of the five assemblage AII isolates we analyzed two additional chromosomal regions: one locus located on chromosome 3 and one on chromosome 5 [28]. Sequences from the assemblage AII isolate JH (chromosome 3 locus (EU188624) and chromosome 5 locus (EU188636)) were used as reference for comparative sequence analyses. Sequencing of the five assemblage AII samples at the chromosome 3 locus yielded three subgroups, where one showed a pattern identical to isolate 335 (GU1119), another one was identical to isolate 303 (GU1086), and a third one gave rise to a unique pattern (GU459, Fig. 3 and Supplementary Table S3). None of the sequences were identical to the JH reference sequence (Fig. 3). Sequencing of the assemblage AII samples at the chromosome 5 locus yielded two different subgroups; one was identical to the JH reference strain and a second identical to isolate 303 (Supplementary Table S4). Interestingly, these two subgroups do not show any correspondence to the groups identified in the chromosome 3 locus (Fig. 3). This suggests again different evolutionary histories of the two loci studied, which could be the result of recombination between sub-assemblage AII isolates.

### *Giardia* assemblage and *H. pylori* colonization

We studied if any of the two human *Giardia* assemblages (A and B) could be specifically associated to *H. pylori* colonization. *Giardia* assemblage B infection had a weak association with *H. pylori* colonization with an Odds Ratio with 95% Confidence interval (OR 95%CI) of 5.0 (1.9–16), Table 2, but more data are needed in order to claim an assemblage-specific association with *H. pylori*. We also noticed that females mainly harbored assemblage A and males assemblage B (Supplementary Table S5). Mixed assemblage infections were only seen in the 1<5 age group and assemblage B dominate in the 5<12 age group (data not shown).

**Table 2:** *Giardia intestinalis* genotypes and association in *H. pylori* positive children from Kampala, Uganda.

*G. intestinalis* assemblage	NumberN	*H. pylori* positive n	Odds Ratio (95% Confidence interval)
**A**	5	3	2.4 (0.3–29)
**B**	25	19	5.0 (1.9–16)
**A+B**	4	2	1.6 (0.1–22)
**Not typed**	52	33	2.7 (1.5–5.3)
**Negative**	341	132	1.0
**Total**	427	189	-

## Discussion

This is a multi-locus genotyping study of *G. intestinalis* isolates from apparently healthy children in an urban setting in Eastern Africa, south of Sahara (Kampala, Uganda). The overall prevalence of *G. intestinalis* in the study population was 20%, which is lower than in earlier studies in rural districts in Uganda [19]. In the study population, we found that children aged 1<5 years had the highest frequency of giardial infection. These findings correlate with the report from in a survey from rural West-Uganda, where a higher rate of colonization was described in children as compared to adults [19] and this is most likely due to unsanitary conditions in these settings. It is important to note here that even if all children enrolled in this study were apparently healthy they constitute a reservoir for transmission of the disease to other children. As indicated in Table 1, there was no statistically significant association between *G. intestinalis* and gender, type of toilet, source of drinking water or type of housing, for the study population, which is indicative of a broad presence of *Giardia* in the environment where these children reside. Berkman et al, previously reported that *Giardia* infections may lead to complications such as; stunted growth, poor cognitive function, and sometimes death, in young children [10], which highlights the significance of our findings and suggests further efforts are needed regarding the prevention of transmission of *Giardia* and other gastro-intestinal pathogens in young children in low-income countries.

The two different *Giardia* assemblages A and B have been associated with different symptoms [15], [16]. Here we have shown that children in urban Kampala, Uganda, predominantly carry assemblage B *Giardia*, which conforms well to reports from several other studied regions of the world [15], [16], [23], [32], [33], [34] but it differs compared to an earlier genotyping study in Uganda [19]. This suggests large local differences in the prevalences of different *Giardia* assemblages. The genetic diversity of the *Giardia* assemblage B lineages present in our study area in Uganda is very high, comparable to the genetic diversity observed among animals and tourists infected in different places in the world (Fig. 1) [13], [16]. Genotyping of *Giardia* isolates is problematic. Several recent reports have shed light on the presence of highly frequent, ambiguous, substitution patterns, which are visualized as double peaks within single nucleotide positions in the sequencing chromatograms upon performing molecular sequence analysis of *Giardia* isolates [13], [16], [23]. It has previously been suggested that these sequence substitutions are due to mixed sub-assemblage infections, allelic sequence heterozygosity (ASH), or a mixture of the two within a patient sample [16], [23], [35], [36]. It should be noted here that *Giardia* is an unusual eukaryote with two diploid nuclei, thus there are at least four different alleles of each gene. Accumulated data suggest that recombination occur between different *Giardia* isolates [37]. A high transmission rate, which is common in endemic areas, could potentially lead to a higher rate of exchange of genetic material between different isolates, which in turn could increase the problems with typing and phylogenetic analyses, as seen here. It should, however, be noted that these problems do not rule out the use of molecular epidemiology as a tool during *Giardia* outbreaks. However, it is clear that better typing methodology is needed.

We found a significantly higher frequency (3 times) of giardial infection in cases where infected children also harbored the bacterial pathogen *H. pylori*. This is comparable with findings from recently published cross-sectional surveys [2], [3], [4]. We also found a weak but specific link to *Giardia* assemblage B (Table 2). Transmission of *H. pylori* is not completely clear [38] but it is possible that the two infectious diseases are transmitted via the same route; the fecal-oral route, and that this explains the high level co-infections. It has been suggested that *H. pylori* transmission in low resource settings is more complex (transmission via food, water and non-parental caretakers to infant) than in high-income countries where within-family transmission seem to dominate [38]. It will be interesting to study the genetic variability of *H. pylori* and *G. intestinalis* isolates within families in our study area since this can answer questions about transmission and establishment of co-infections. This will be important in the development of measures to reduce transmission of these two important pathogens.

The importance of polymicrobial infections has gained tremendous impact in recent years and synergistic infections have been identified [39]. In synergistic polymicrobial infections, one microbe creates a favorable environment in order for another one to more easily colonize a specific niche of their common host [39]. *H. pylori* has been linked to co-infections earlier, e.g. the fluke *Schistosoma japonicum* is associated with an alteration in the antibody response to *H. pylori* during co-infections [40]. Another interesting example is co-infections of *H. pylori* and *Salmonella typhimurium* in mice [41]. In this study it was shown that *H. pylori* represses the Th17 response in the lower gastrointestinal (GI) tract via extragastric immunomodulatory factors. Increased IL-10 expression was seen in mesenteric lymph nodes and in the cecum. Regulatory T cells activated by *H. pylori* in the stomach have been shown to reduce inflammation in the lungs and to prevent induction of allergic asthma [42]. This shows that *H. pylori* infection in the stomach can induce immunoregulatory responses systemically and also in the intestine. The large number of co-infections in our study is possibly due to an elevated risk of *G. intestinalis* colonization upon the presence of *H. pylori* in human patients or, alternatively, *H. pylori* colonization may be facilitated by a previous establishment of *Giardia*. A longitudinal study of children from birth to 3 years of age were *Giardia* and *H.pylori* infections are diagnosed monthly and during diarrhea episodes could resolve this issue and also show if symptoms are affected by the other infection. The mechanisms behind this potential microbial interplay indeed need to be further investigated. Since both pathogens may be cultured *in vitro* [43], [44] as well as they both successfully infect gerbils [45], [46], *in vitro* and *in vivo* assays may be implemented to gain further understanding of how they interact with the host's immune response but also to determine if *H. pylori*-induced changes in the pH level of the stomach facilitate *Giardia* infections [47].

In conclusion, 20% of the 427 healthy children in this region of urban Kampala, Uganda were carriers of *Giardia*. Molecular sequence analysis of a sub-set of the *Giardia* positive samples (n = 45) showed 15% assemblage A, 74% assemblage B, and 11% mixed A and B assemblage infections. The genetic variability was very high in the assemblage B isolates, whereas it was low in assemblage A isolates. We found a strong correlation of concomitant *G. intestinalis* and *H. pylori* infections in children in Kampala, Uganda and a weak association to *Giardia* assemblage B. This information will be important in the design of further studies of these pathogens in Uganda and other low-income countries in order to develop new control measures.

## Supporting Information

File S1 Oligonucleotides used in PCR analysis.
(DOCX)

File S2 Sequences from the beta-giardin locus from all positive samples.
(TXT)

File S3 Sequences from the glutamate dehydrogenase locus from all positive samples.
(TXT)

File S4 Sequences from the triose-phosphate isomerase locus from all positive samples.
(TXT)

Table S1 Distribution of *Giardia* assemblages established by nested-PCR and sequencing of the *bg*, *tpi* and *gdh* loci, compiled with assemblage A- and B-specific *tpi* PCR.
(DOCX)

Table S2 Mixed assemblage A and B infection at each genetic locus, including the results from the PCR where assemblage specific primers where utilized.
(DOCX)

Table S3 Characterization *G. intestinalis* assemblage A from children in Kampala, Uganda at the chromosome 3 SNP locus.
(DOCX)

Table S4 Characterization *G. intestinalis* assemblage A from children in Kampala, Uganda at the chromosome 5 SNP locus.
(DOCX)

Table S5 Age and gender distribution by different genotype of Giardia.
(DOCX)
