# Supplementary material for: Common Coinfections of Giardia intestinalis and Helicobacter pylori in Non-Symptomatic Ugandan Children
Source: PLoS Negl Trop Dis. 2012 Aug 28;6(8):e1780. doi: 10.1371/journal.pntd.0001780 (PMC3429385; doi:10.1371/journal.pntd.0001780)
Supplement: File S1 — Oligonucleotides used in PCR analysis. (DOCX) [file pntd.0001780.s001.docx]

**Supplementary File 1: *G. intestinalis H. pylori* Uganda**

| **Locus** | **Primer sequences** |
| --- | --- |
| ***bg*** (Cacciò et al., 2002)  (Lalle et al., 2005) | Fwd1: 5′AAGCCCGACGACCTCACCCGCAGTGC-3′  Rev1: 5′GAGGCCGCCCTGGATCTTCGAGACGAC-3′  Fwd2: 5′-GAACGAGATCGAGGTCCG**-**3′ Rev2: 5′-CTCGACGAGCTTCGTGTT**-**3′ |
| ***gdh*** (Read et al., 2004) | Fwd1: 5’-TCAACGTYAAYCGYGGYTTCCGT-3’ Rev1&2: 5’-GTTRTCCTTGCACATCTCC-3’  Fwd2: 5’-CAGTACAACTCYGCTCTCGG-3’ |
| ***tpi***  (Sulaiman et al., 2003) | Fwd1: 5′-AAATIATGCCTGCTCGTCG-3′ Rev1: 5′-CAAACCTTITCCGCAAACC-3′  Fwd2: 5′-CCCTTCATCGGIGGTAACTT-3′ Rev2: 5′-GTGGCCACCACICCCGTGCC-3′ |
| ***tpi*** (Ass A specific)  (Geurden et al., 2007) | Fwd2: 5′-CGCCGTACACCTGTCA-3′ Rev2: 5′-AGC AAT GAC AAC CTC CTT CC-3′ |
| ***tpi*** (Ass B specific)  (Geurden et al., 2009) | Fwd2: 5′-GTTGTTGTTGCTCCCTCCTTT-3′ Rev2: 5′-CCGGCTCATAGGCAATTACA-3′ |
| **Chromosome 3**  (Cooper et al., 2010) | Fwd1: 5’-TGGAGGCGGTCAAGATACTC-3’ Rev1: 5’-CTCGACGATTATGCTCCACGACG-3’   Fwd2: 5’-CGGTCAAGATACTCTACGATCG-3’ Rev2: 5’-CTCGACGATTATGCTCCACGACG-3’ |
| **Chromosome 5**  (Cooper et al., 2010) | Fwd1: 5’GGCGAGTGCAGTCCTGAGTGG-3’ Rev1: 5’CCTGGCTTGTTAACTACATCC3’  Fwd2: 5’-CAGTTTGGAAGAGCAGGACTCG-3’ Rev2: 5’-GTCATCTTTCTATGCCTTCTTCG-3’ |
