# Supplementary material for: Common Coinfections of Giardia intestinalis and Helicobacter pylori in Non-Symptomatic Ugandan Children
Source: PLoS Negl Trop Dis. 2012 Aug 28;6(8):e1780. doi: 10.1371/journal.pntd.0001780 (PMC3429385; doi:10.1371/journal.pntd.0001780)
Supplement: Table S1 — Distribution of Giardia assemblages established by nested-PCR and sequencing of the bg, tpi and gdh loci, compiled with assemblage A- and B-specific tpi PCR. (DOCX) [file pntd.0001780.s005.docx]

**Supplementary Table 1.** Distribution of *Giardia* assemblages established by nested-PCR and sequencing of the *bg*, *tpi* and *gdh* loci, compiled with assemblage A- and B-specific *tpi* PCR

|  | **A**  **n** | **B**  **n** | **A+B**  **n** | **Not Amplified**  **n** | **Total Positives**  **N** |
| --- | --- | --- | --- | --- | --- |
| *bg* | 5 | 26 | 1 | 16 | 32 |
| ***gdh*** | 5 | 28 | 1 | 13 | 34 |
| ***tpi*** | 3 | 25 | 1 | 18 | 29 |
| ***tpi* (assemblage specific PCR)** | 5 | 25 | 4 | 15 | 34 |
| **Combined Results** | 5 | 25 | 4 | 13 | 34 |
