# Supplementary material for: Common Coinfections of Giardia intestinalis and Helicobacter pylori in Non-Symptomatic Ugandan Children
Source: PLoS Negl Trop Dis. 2012 Aug 28;6(8):e1780. doi: 10.1371/journal.pntd.0001780 (PMC3429385; doi:10.1371/journal.pntd.0001780)
Supplement: Table S2 — Mixed assemblage A and B infection at each genetic locus, including the results from the PCR where assemblage specific primers where utilized. (DOCX) [file pntd.0001780.s006.docx]

**Supplementary Table 2.** Mixed assemblage A and B infection at each genetic locus, including the results from the PCR where assemblage specific primers where utilized

| **Isolate** | ***bg*** | ***gdh*** | ***tpi*** | ***tpi* ass. spec. PCR** |
| --- | --- | --- | --- | --- |
| **UG321** | B | B | B | A+B |
| **UG1046** | B | B | B | A+B |
| **UG1073** | B | B | B | A+B |
| **UG1165** | A+B | A+B | A+B | A+B |
