# Supplementary material for: Common Coinfections of Giardia intestinalis and Helicobacter pylori in Non-Symptomatic Ugandan Children
Source: PLoS Negl Trop Dis. 2012 Aug 28;6(8):e1780. doi: 10.1371/journal.pntd.0001780 (PMC3429385; doi:10.1371/journal.pntd.0001780)
Supplement: Table S3 — Characterization G. intestinalis assemblage A from children in Kampala, Uganda at the chromosome 3 SNP locus. (DOCX) [file pntd.0001780.s007.docx]

**Supplementary Table 3.** Characterization *G. intestinalis* assemblage A from children in Kampala, Uganda at the chromosome 3 SNP locus

| **AII Isolates** | **20** | **26** | **195** | **216** | **317** | **396** |
| --- | --- | --- | --- | --- | --- | --- |
| **JH** | G | T | G | A | G | T |
| **335** | * | * | * | G | * | * |
| GU436, GU1116, GU1119 | * | * | * | G | * | * |
| **303** | A | C | A | G | * | * |
| GU1086 | A | C | A | G | * | * |
| GU459^*^ | * | * | * | G | A | * |

**Note.** JH (sub-assemblage AII) is used as a baseline, sequences from isolates 303 and 335 come from Cooper et al., 2010.

^*^ GU459 gave rise to a new sequence pattern as compared to earlier published sequences at the chromosome 3 SNP locus.
