# Supplementary material for: Common Coinfections of Giardia intestinalis and Helicobacter pylori in Non-Symptomatic Ugandan Children
Source: PLoS Negl Trop Dis. 2012 Aug 28;6(8):e1780. doi: 10.1371/journal.pntd.0001780 (PMC3429385; doi:10.1371/journal.pntd.0001780)
Supplement: Table S4 — Characterization G. intestinalis assemblage A from children in Kampala, Uganda at the chromosome 5 SNP locus. (DOCX) [file pntd.0001780.s008.docx]

**Supplementary Table 4.** Characterization *G. intestinalis* assemblage A from children in Kampala, Uganda at the chromosome 5 SNP locus

| **AII Isolates** | **29** | **156** | **212** | **234** | **282** | **477** | **482** |
| --- | --- | --- | --- | --- | --- | --- | --- |
| **JH** | A | G | C | T | C | A | G |
| GU1086, GU1116 | * | * | * | * | * | * | * |
| **303** | * | * | T | C | * | G | A |
| GU436, GU459, GU1119 | * | * | T | C | * | G | A |

**Note.** JH (sub-assemblage AII) is used as a baseline, sequences from isolate 303 come from Cooper et al., 2010.
