# Supplementary material for: Common Coinfections of Giardia intestinalis and Helicobacter pylori in Non-Symptomatic Ugandan Children
Source: PLoS Negl Trop Dis. 2012 Aug 28;6(8):e1780. doi: 10.1371/journal.pntd.0001780 (PMC3429385; doi:10.1371/journal.pntd.0001780)
Supplement: Table S5 — Age and gender distribution by different genotype of Giardia. (DOCX) [file pntd.0001780.s009.docx]

**Supplementary Table 5:** Age and gender distribution by different genotype of Giardia.

|  | Type A  N (%) | Type B  N (%) | Mix A and B  N (%) | Giardia found by direct microscopy  N (%) |
| --- | --- | --- | --- | --- |
| Female | 4 (80.0) | 10 (40.0) | 2 (50.0) | 41 (19.0) |
| Male | 1 (20.0) | 15 (60.0) | 2 (50.0) | 45 (21.3) |
| Age (± SD) | 5.1 (±1.9) | 5.5(±3.2) | 3.7(±0) |  |
